# Delay in detection of urethral catheter misplacement in the vagina of an older adult patient due to urinary outflow during catheterization: a case report

**DOI:** 10.1186/s40981-025-00776-x

**Published:** 2025-02-28

**Authors:** Hisashi Shio, Takao Setsu, Tomonobu Takagaki, Hiroshi Adachi, Makoto Kiuchi, Hideyuki Tsubokura

**Affiliations:** 1 Department of Anesthesiology, Tottori Red Cross Hospital, 117 Shotoku-Cho, Tottori City, Tottori 680-8517 Japan; 2 Department of Obstetrics and Gynecology, Tottori Red Cross Hospital, Tottori City, 117 Shotoku-Cho, Tottori 680-8517 Japan

**Keywords:** Older adult patient, Female urethral catheterization, Difficult urethral catheterization, Uretheral catheter misplacement in the vagina, Vesicovaginal reflux, Urinary incontinence

## Abstract

**Background:**

To our knowledge, no previous case report explicitly shows that urethral catheter misplacement in the vagina cannot be ruled out even if urinary outflow is observed during catheterization.

**Case presentation:**

A 70-year-old female underwent urethral catheterization during induction of general anesthesia for hemiarthroplasty using a bipolar hip prosthesis. Although the urethral meatus could not be visualized, urinary outflow was observed. However, drainage subsequently stopped, and the catheter was eventually found to have been misplaced in the vagina. Detection of the catheter misplacement was delayed because of the assumption that no urinary outflow occurs when the catheter is misplaced in the vagina.

**Conclusion:**

Even if urinary outflow is observed during female urethral catheterization, catheter misplacement in the vagina cannot be ruled out; therefore, catheter misplacement in the vagina must be verified in patients for whom the urethral meatus cannot be identified for catheter insertion or when drainage stops.

## Background

Urethral catheterization is commonly performed during the perioperative period. Previous literature includes detailed procedures and troubleshooting for safe, accurate placement of urethral catheters [1–5]. However, to our knowledge, no previous reports have explicitly described cases in which urethral catheter misplacement in the vagina cannot be ruled out, even if urinary outflow is observed. We report the case of an older adult patient in whom a urethral catheter was mistakenly placed in the vagina. Recognition of this issue was delayed because urinary outflow was observed during catheterization.

## Case presentation

The patient was a 70-year-old female (height, 150 cm; body weight, 53 kg) who fell and sustained a right femoral neck fracture in the morning. A hemiarthroplasty using a bipolar hip prosthesis was planned for the evening of the same day as the injury. The patient had a history of head trauma and ventriculoperitoneal shunt surgery 10 years prior to presentation. Although the patient was able to communicate and walk with a walker, functional urinary incontinence was experienced daily because of slow movement. Preoperative laboratory data revealed renal dysfunction (estimated glomerular filtration rate, 41.2 ml/min/1.73 m^2^) and an elevated D-dimer of 46.4 µg/ml. Blood gas analysis showed PaO2 of 57.2 mmHg at room air, although the causes of hypoxia were not detected by plain and contrast-enhanced chest computed tomography or transthoracic echocardiography.

**Fig. 1:**
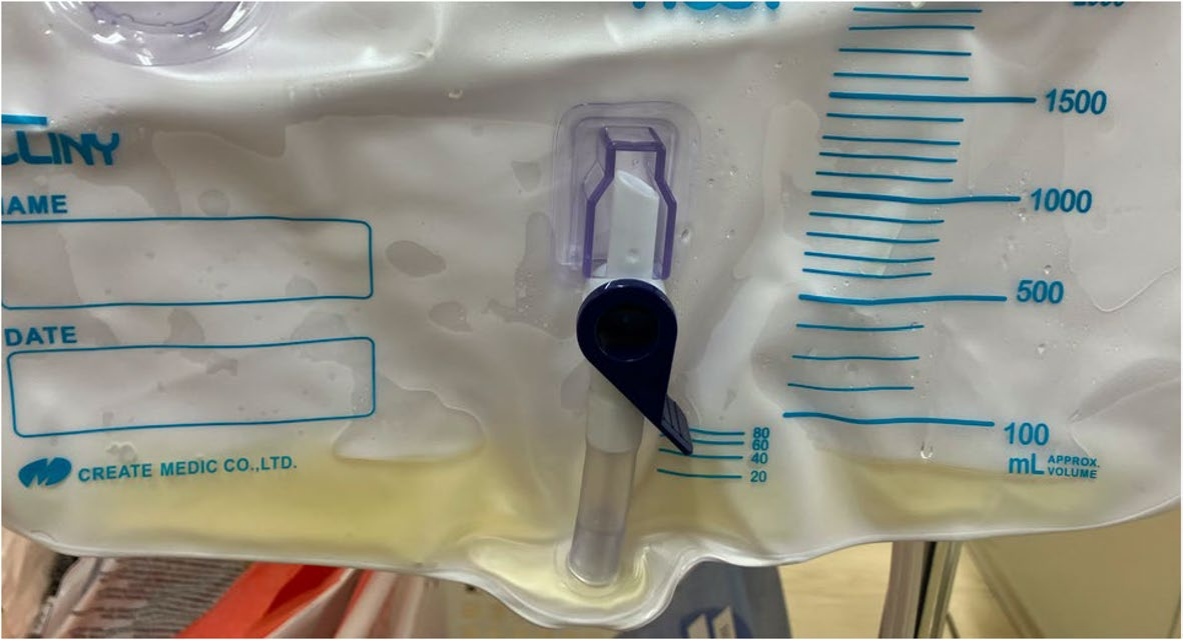
Appearance of the urinary drainage bag after surgery. Approximately, 20 ml of pale-yellow fluid was collected before surgery, with no additional discharge during surgery

General anesthesia and nerve blocks were planned for surgery. After induction of general anesthesia, the patient was placed in the left lateral position for surgery. Immediately after sedation was achieved and the patient was unconscious, two nurses attempted to place a 12-Fr all silicone Foley balloon catheter (Create Medic Co., Ltd., Kanagawa, Japan) with the patient in the supine frog-legged position, i.e., supine position with knees flexed upward and the thighs are slightly externally rotated — outward, with limited leg abduction, considering the hip fracture. The urethral meatus could not be visualized; however, upon insertion, fluid consistent with urine flowed spontaneously through the catheter. The nurses assumed that the catheter had been correctly inserted into the bladder and completed the catheterization procedure after confirming unrestricted balloon inflation and the presence of resistance when the catheter was gently pulled. Immediately before surgery, the anesthesiologist observed 20 ml of pale-yellow fluid in the urinary drainage bag (Fig. 1).

The surgery was completed as scheduled, and no obvious respiratory or circulatory problems were observed. General anesthesia was administered for a total of 2 and a half hours. Overall, 1350 ml of isotonic crystalloid solution was infused; 180 g of blood was lost during surgery. The volume in the urinary drainage bag remained at 20 ml; no additional fluid was obtained even when the catheter was aspirated with a syringe. The anesthesiologist recognized the cessation of catheter drainage as a problem, however did not suspect catheter misplacement in the vagina because of the presence of drainage fluid consistent with urine.

**Fig. 2:**
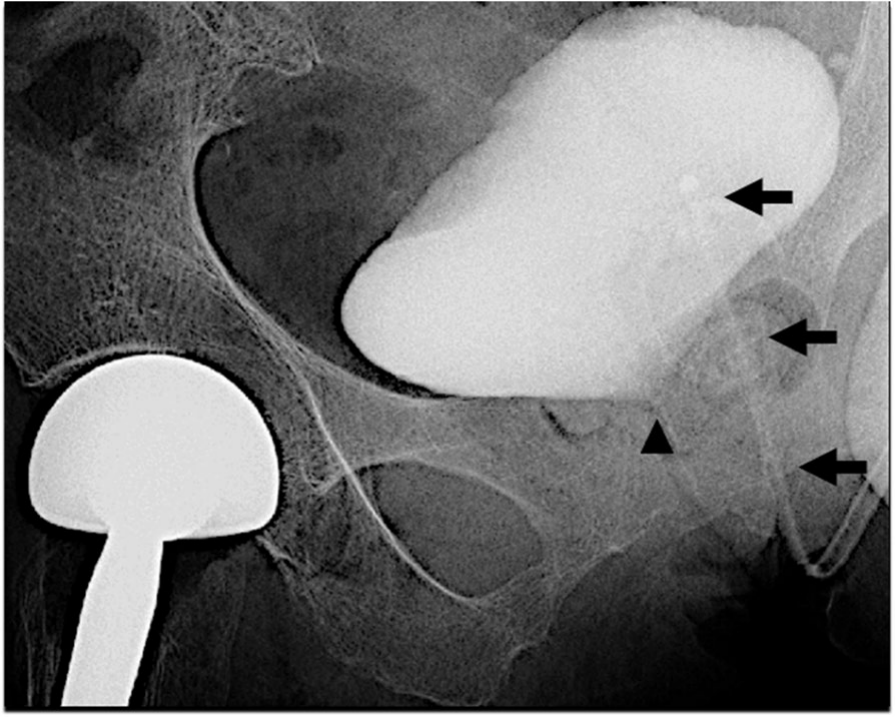
Postoperative right hip radiograph. Lauenstein projection showed a urethral catheter (arrow) not passing through the internal urethral orifice (arrowhead) with retained urine containing contrast medium in the bladder

**Fig. 3:**
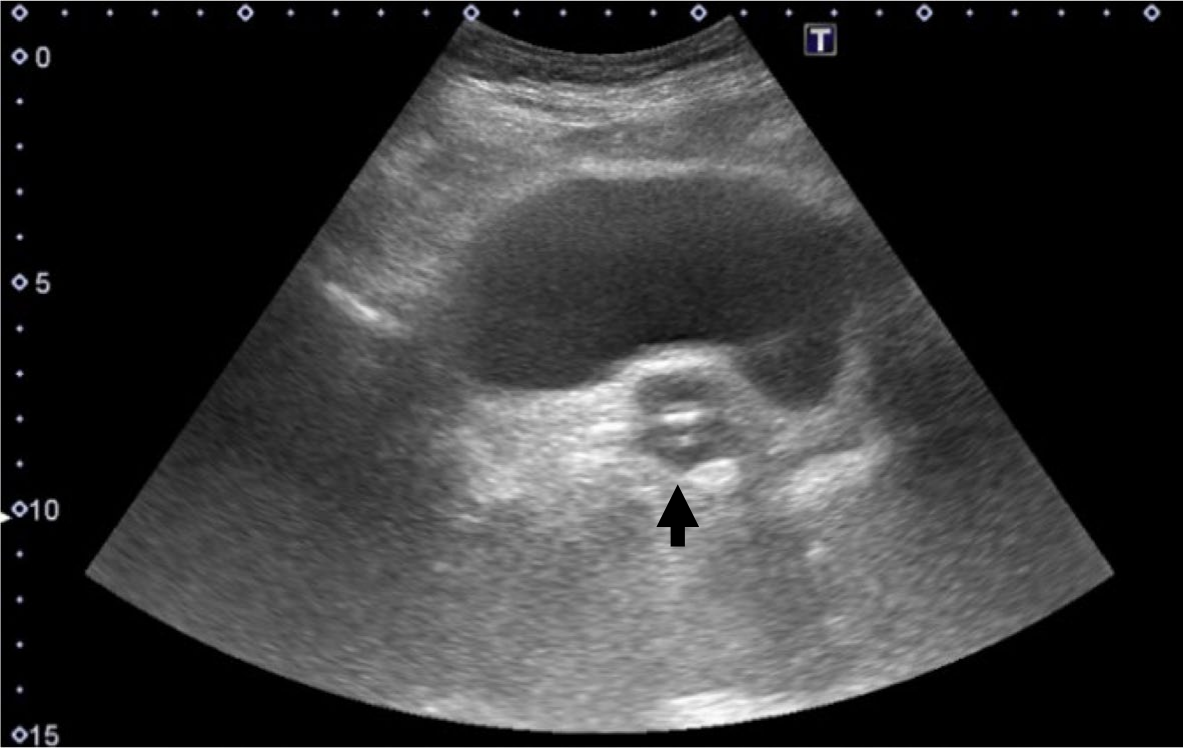
Transverse ultrasound image of the bladder showing a urethral catheter balloon (arrow) on the dorsal side of the bladder

As no additional drainage occurred 30 min after transfer to the high-care unit, laboratory tests and treatments were planned for acute kidney injury, with contrast-induced nephropathy considered as the most likely cause. At that time, the anesthesiologist noticed that a postoperative radiograph of the right hip showed urine mixed with contrast in the bladder, and that the catheter had not passed through the internal urethral orifice (Fig. 2), indicating catheter misplacement. A urethral catheter balloon was identified on the dorsal side of the bladder on bladder ultrasound (Fig. 3). Catheter misplacement in the vagina was confirmed on pelvic examination, therefore precipitating removal.

When recatheterization was attempted, the urethral meatus could not be visualized with the patient in the same frog-legged position with limited leg abduction. However, recatheterization was successful because the location of the meatus was estimated using palpation near the vaginal opening. Immediately after recatheterization, 400 ml of urine was collected, and the appearance of the fluid was nearly identical to that observed after the initial catheterization. After consulting an obstetric gynecologist regarding the drainage fluid after the initial catheterization, the fluid was determined to be urine and not vaginal discharge, based on volume and appearance.

Several days later, a physical examination was performed by an obstetricic gynecologist. The urethral meatus was visible adjacent to the vaginal opening when the patient assumed the lithotomy position for examination. No obvious anatomical abnormalities were observed other than postmenopausal urogenital atrophy.

## Discussion

The female urethra is short, approximately 3.5 to 5 cm [1, 2], and insertion of a urethral catheter is generally easier in women than in men when the urethral meatus can be visualized [2]. However, the meatus is often difficult to visualize, which can lead to catheter misplacement in the vagina [1–3]. The absence of urinary outflow is an indication that a catheter may be in the vagina [2, 3]. However, in the present patient, catheter misplacement was not detected in a timely manner because of the assumption that had the catheter been misplaced in the vagina, no urinary outflow would have occurred. Had the problem not been discovered in our patient, unnecessary laboratory tests and treatments would have been conducted to address the subsequent lack of urinary output. Therefore, this patient represents a didactic case illustrating that even if urinary outflow is observed during female urethral catheterization, catheter misplacement in the vagina cannot be ruled out. This potential issue cannot be ignored by anesthesiologists who use urinary output for perioperative management. Even if urinary outflow is observed during female urethral catheterization, if the urethral meatus cannot be identified upon catheter insertion, or if drainage stops, catheter misplacement in the vagina must be ruled out or verified by pelvic examination or bladder ultrasound.

After discovering that the urethral catheter has been placed in the vagina in the patient, we considered the possibility that vaginal discharge which was characteristic of urine had drained. However, vaginal discharge in older adults is normally minimal unless vaginitis is present, which causes increased purulent vaginal discharge [6]. After consulting an obstetric gynecologist, we confirmed the drainage fluid to be urine and not vaginal discharge based on the large volume and pale-yellow appearance.

This led us to ask the following question. Why was urine retained in the vagina at the time of urethral catheterization? Reportedly, a urogenital fistula (UGF) [7, 8] and vesicovaginal reflux (VVR) [9–11] can cause urine to enter the vagina. Our patient had no history of urinary incontinence suspected to be congenital UGF and no history of pelvic surgery, malignancy, or radiotherapy that could have caused acquired UGF [7, 8]. In addition, the absence of persistent urinary outflow during the initial urethral catheterization essentially ruled out UGF. In contrast, VVR is defined as the reflux of urine into the vaginal vault during voiding [9] and has been reported as an underestimated cause of urinary incontinence in adult females [10]. Labial adhesions or tightly opposed labia majora may cause backflow into the vagina by preventing the passage of urine [11]. In the present case, upon removal of the second urethral catheter, the tightly opposed labia majora prevented complete voiding in the supine position in which the legs were in a neutral position (not abducted), and the urine flowed down the perineal and buttock skin. In addition, the patient experienced functional urinary incontinence daily and remained in the supine position with the legs in neutral after the fracture. Therefore, VVR likely occurred during voiding while the patient was in this position, and urine remained in the vagina because the patient remained in the position until surgery.

Moreover, we recognize human error in that the urethral catheter was misplaced in the vagina because of catheter insertion under conditions in which visualizing the urethral meatus was difficult. This difficulty may have been precipitated by the anatomical shift of the meatus to the vicinity of the vaginal opening because of postmenopausal vaginal atrophy [5] and inadequate positioning during catheter insertion. The patient was under general anesthesia; therefore, an adequate supine frog-legged or lithotomy position might have been a better choice for visualization of the meatus. In cases where the meatus is difficult to visualize despite efforts to adjust positioning and lighting, proper catheter insertion can be managed by *the clinician* inserting a finger into the vagina as a guide [1, 2, 4, 5]. In addition, bladder ultrasound is easy to perform in the operating room and useful for confirming the correct position of the urethral catheter in the bladder [12]. These measures would help prevent catheter misplacement and ensure correct catheter placement. If multiple catheter insertion attempts fail, a urologist or gynecologist should be consulted.

In summary, we encountered an older adult patient in whom the urethral catheter was misplaced in the vagina. Detection was delayed because urinary outflow had been observed during the catheterization. Even if urinary outflow is observed during female urethral catheterization, catheter misplacement in the vagina cannot be ruled out. Therefore, catheter misplacement in the vagina must be ruled out in cases in which the urethral meatus cannot be identified upon insertion of the catheter or drainage stops.

## Data Availability

Data relevant to this case report are not available for public access because of patient privacy concerns; however, these data are available from the corresponding author upon reasonable request.

## Abbreviations

UGF: Urogenital fistula
VVR: Vesicovaginal reflux
